# No evidence for theta power as a marker of hypnotic state in highly hypnotizable subjects

**DOI:** 10.1016/j.heliyon.2021.e06871

**Published:** 2021-04-29

**Authors:** Seppo Hiltunen, Maria Karevaara, Maarit Virta, Tommi Makkonen, Sakari Kallio, Petri Paavilainen

**Affiliations:** a Teaching and Learning Services, University Services, University of Helsinki, Finland; b Department of Psychology and Logopedics, Faculty of Medicine, University of Helsinki, Finland; c Department of Cognitive Neuroscience and Philosophy, School of Bioscience, University of Skövde, Sweden; d Centre for Cognitive Neuroscience, University of Turku, Finland; e Cognitive Brain Research Unit, Department of Psychology and Logopedics, University of Helsinki, Finland

**Keywords:** Hypnosis, Hypnotic suggestion, Brain oscillations, Spectral-power density, EEG

## Abstract

EEG spectral-power density was analyzed in a group of nine highly hypnotizable subjects via ten frontal, central, parietal, and occipital electrodes under four conditions: 1) wake state, 2) neutral hypnosis, 3) hypnotic suggestion for altering perception of tones, and 4) post-hypnosis. Results indicate no theta-power changes between conditions, challenging previous findings that increased theta power is a marker of hypnosis. A decrease in gamma power under hypnotic suggestion and an almost significant decrease under neutral hypnosis were observed, compared to post-hypnosis. Anteroposterior power distribution remained stable over all conditions. The results are discussed and compared to earlier studies, which report heterogenous findings.

## 1 Introduction

The search for the neural correlates of hypnosis and hypnotic suggestion has been of continuing interest to the neuroscience of hypnosis (Halsband and Wolf, 2019). The specific top-down cognitive mechanisms underlying responsiveness to hypnotic suggestion have nevertheless remained poorly understood (Terhune et al., 2017). Whether or not hypnosis should be understood as an altered state of consciousness has long been debated (e.g., Kallio and Revonsuo, 2003; Kihlstrom, 2005; Kirsch and Lynn, 1995); even the very relevance of hypnosis to altered states of consciousness studies has been questioned (Terhune et al., 2017). Though studies using electrical brain oscillations as potential indices of hypnosis began decades ago (Galbraith et al., 1970; Tebecis et al., 1975), modern multichannel electroencephalography (EEG) may still provide additional information about the relevant neural processes.

The EEG frequency domain has traditionally been divided into delta (1–4 Hz), theta (4–8 Hz), alpha (8–13 Hz), beta (13–25 Hz) and gamma (>25 Hz) bands. Several sub-bands have typically been used (theta1, theta2, alpha1, alpha2 etc.). Possible hypnosis-related oscillatory power changes in EEG have been a focus of interest for decades. Changes have been reported in all EEG bands. That said, a review of brain-oscillation studies of hypnosis (Jensen et al., 2015) found hypnosis to be linked most consistently to increase in theta power and possibly also changes in gamma activity.

Both with high and low hypnotizable subjects, hypnosis has been found to increase mean theta power (Sabourin et al., 1990; Williams and Gruzelier, 2001). Compared to low hypnotizable subjects in wake state, highly hypnotizable subjects have exhibited more theta power across many studies (Graffin et al., 1995; Kirenskaya et al., 2011; Vanhaudenhuyse et al., 2014), even as other studies have found no theta differences between the groups (De Pascalis, 1999). Further research on the role of theta oscillations as indicator of hypnotizability is warranted (Terhune et al., 2017).

In non-hypnosis conditions, theta oscillations have been associated with such overt and covert behaviors as orientation, attention, voluntary movement, working memory, and general memory encoding and retrieval (for details, see Buzsaki, 2005). Theta power typically varies together with cognitive performance on memory tasks (Buzsaki, 2006).

Gamma oscillations (>25 Hz) are widely distributed through the brain, reflecting the diverse functions of the nervous system (Basar, 2013); both their power and level of variance can vary extensively (Buzsaki, 2006). Gamma oscillations are typically modulated by internal processes such as working-memory operation, selective attention, and (in particular) sensory input (Jia and Kohn, 2011). Narrow-band gamma (~20–60 Hz), which can be studied with standard EEG methodology, is elicited by simple sensory stimuli (Bartoli et al., 2019); the oscillations are often thought to be related to conscious perception of such stimuli (Jia and Kohn, 2011). Gamma-band responses have been observed to reflect purely visual sensory input, which seems to challenge their relation to conscious perception in higher-order visual cortices (Aru et al., 2012; Pitts et al., 2014). Gamma-band responses appear dependent on the attributes and class of the presented stimuli.

Early hypnosis studies found that alpha oscillations increased during hypnosis (Graffin et al., 1995; Lubar et al., 1991); but the effect seems to have been more related to closing the eyes and relaxing than to hypnosis *per se*. Some more recent studies have not shown hypnosis-related power changes in the theta or alpha bands (White et al., 2008) or in *any* EEG frequency bands (Jamieson and Burgess, 2014).

Hypnosis was associated with decreased delta and increased beta activity in the frontal areas of a hypnotic virtuoso, but the observed changes were found mainly in the composition of brain oscillations rather than oscillatory power (Fingelkurts et al., 2007). In highly hypnotizable subjects, self reported depth of hypnosis has been found to correlate with power in the beta and gamma bands (Cardeña et al., 2013).

Oscillatory power changes associated with specific hypnotic suggestions intended to alter a highly hypnotizable subject's perceptual, motor, or cognitive processes have been studied as well. Gamma activity has been shown to be influenced by hypnosis and hypnotic suggestions (e.g., De Pascalis, 1999; De Pascalis et al., 1989; De Pascalis, Ray, Tranquillo & D'Amigo, 1998). A lateralized 40 Hz gamma-power effect during emotional recall (decrease in the left hemisphere with negative emotions, increase in both hemispheres with positive emotions) has been observed (De Pascalis et al., 1989). The direction of effects has not been consistent between studies, however, and may depend on factors related to the nature of the suggestions, experimental setup, or EEG-measurement parameters (Jensen et al., 2015).

A decrease in posterior alpha power during hypnotic dreaming and age-regression suggestions and an increase in the left-hemisphere beta power during age regression have been reported (De Pascalis, 1993). One study compared oscillatory activity between self-generated happy and sad emotions during hypnosis; sad emotions showed significantly less alpha activity in the right parietal areas (Crawford et al., 1996).

Taken together, research findings concerning the EEG correlates of hypnotizability, hypnotic induction, and hypnotic suggestion have been heterogenous: which is to say, inconsistent and difficult to interpret (Cardeña et al., 2013; Halsband and Wolf, 2019). The present study aims to clarify whether the oscillatory power of various EEG frequency bands differs in highly hypnotizable subjects between wake state (pre-hypnosis), neutral hypnosis, hypnosis with hypnotic suggestions, and post-hypnosis – with particular interest in theta and gamma activity, considered by Jensen et al. (2015) as being most consistently linked to hypnosis.

We used the EEG data from our earlier study (Hiltunen et al., 2019), which focused on whether hypnosis and hypnotic suggestion have an effect on the pre-attentive mismatch negativity (MMN) component of the event-related potential (ERP). MMN is a fronto-central, relatively automatic, attention-independent negative deflection to an auditory stimulus change. It usually peaks 100–250 ms after onset of occasional deviant stimuli, presented among physically similar “standard” stimuli (Sussman, 2007; Näätänen et al., 2007).

Under all conditions of the earlier study, short standard tones and deviant tones (differing from the standard ones in pitch) were presented in the background at 0.5-second intervals. By giving subjects the suggestion that all auditory stimuli should sound similar in pitch, our study aimed to find out whether the MMN response to the deviant tones could be diminished under hypnosis. Presentation of the background tones could be considered a potential problem in the design of the present study, as the constant auditory stimulation might itself have effects on oscillatory brain activity. However, since the auditory stimulation was identical under all four conditions, its effects on oscillatory brain activity (if any) should be similar.

On the basis of a review of brain oscillations in hypnosis (Jensen et al., 2015), we hypothesized that our highly hypnotizable subjects would exhibit higher theta power under the neutral-hypnosis and hypnotic-suggestion (“all tones sound similar to each other”) conditions, compared to the pre-hypnosis and post-hypnosis conditions (Hypothesis 1). Based on the same review, we hypothesized that gamma power might change (either increase or decrease) under the neutral-hypnosis and hypnotic-suggestion conditions, compared to the pre-hypnosis and post-hypnosis conditions (Hypothesis 2). We further hypothesized that the suggestions intended to alter perception of tones under the hypnotic-suggestion condition might result in a slight change (increase or decrease) in gamma oscillatory power compared to the neutral-hypnosis condition (Hypothesis 3). This last hypothesis was based on Jensen et al. (2015) proposal that implementation of the suggestions might be reflected in changes in gamma activity. Possible changes in alpha, beta, and delta activity were analyzed for exploratory purposes but no hypotheses were put forward.

## 2 Materials and methods

### 2.1 Subjects

Subjects were recruited through advertisements on the mailing lists of psychology and educational-sciences students at the University of Helsinki. Inclusion criteria for participation in the study were (1) that one be 18–45 years of age, (2) with no diagnosis of bipolar disorder or psychosis, (3) having no neurological disorders apart from migraine, and (4) not currently experiencing severe depression. In total, 57 subjects enrolled in the hypnotizability measurement group sessions and 48 participated. Potential subjects completed a questionnaire about work, education, health, and medications. No one was excluded for not meeting the inclusion criteria. Prior to participating, subjects gave their written informed consent.^1^

Hypnotizability of the 48 subjects was measured using the Finnish version (Kallio, 1996; Kallio and Ihamuotila, 1999) of the Harvard Group Scale of Hypnotic Susceptibility, Form A (HGSHS:A) (Shor and Orne, 1962). All of the highly hypnotizable subjects (*N* = 9, score of nine or more on the HGSHS:A) were selected for the EEG measurement session. All subjects were students, and all were right-handed (eight females, one male; mean age: 25.7 years, sd: 5.1, range: 20–37 years; mean education history: 16.1 years, sd: 2.8, range: 12.5–19 years; HGSHS:A mean: 10.1, sd: 0.9, range: 9–11). The mean time between the hypnotizability and EEG measurement sessions was 139.2 days (sd: 61.2, range: 56–243 days).

The study was approved by the University of Helsinki Ethical Review Board in the School of Humanities and Social and Behavioural Sciences. It was performed in accordance with the ethical standards of the Declaration of Helsinki. Subjects participating in the EEG measurement session received as compensation leisure and culture vouchers worth €20.

### 2.2 Stimuli

Auditory and visual stimuli were presented during the EEG recording. Pure, 100-ms sinusoidal tones were used as the auditory stimuli. Under each condition, standard (500 Hz; *p* = .82) and deviant tones (520 Hz, *p* = .18) were presented in random order with a 400-ms interstimulus interval (ISI), with auditory stimulation lasting six minutes and eight seconds per condition. The stimuli were presented from two loudspeakers, positioned to the right and left of the subject. Their intensity was about 56 dB SPL at the subject's ear level.

A silent nature video of a river flowing calmly through a forest was used as visual stimulus. The video was sold commercially for relaxation purposes.^2^ It was shown on an 18-inch display placed in front of the subject.^3^

### 2.3 Procedure

Subjects were seated in a reclining armchair in an acoustically and electrically shielded room. The experimenter (MV), who administered all hypnosis sessions, sat behind and to the right of the subject. Before starting the experiment, the experimenter told the subject that her task under all conditions was simply to watch the video and relax, with no need to pay attention to the tones. The subject was asked to avoid excessive eye blinking during the video, if possible. The four experimental conditions were presented in the following order, with the whole procedure lasting about 45 min^4^

**1) Pre-hypnosis** (**PrH**): The experimenter instructed the subject to watch the video while the auditory stimuli were delivered.

**2) Neutral hypnosis (HY)**: Before presenting the auditory stimuli, the experimenter carried out a hypnotic induction in a structured way, while allowing for some personal modification (e.g., time for closing the eyes). Lasting around eight minutes, the induction consisted of eye fixation, closing of the eyes, relaxation, and deepening of hypnosis through counting. The subject was subsequently asked to open her eyes and start watching the video. The auditory stimulation started simultaneously. Once the condition was fully underway, a few more suggestions for intensifying depth of hypnosis were given. When the condition ended, the subject was asked to close her eyes.

**3) Hypnotic suggestion (SU):** The experimenter began by giving a suggestion to alter the subject's perception of the auditory stimuli. The suggestion was formulated to suggest that all tone beeps sound exactly the same in pitch, played softly in the background and lacking in meaning. The experimenter then asked the subject to open her eyes and watch the video; and he started the auditory stimulus block. Once the condition was underway, he gave a few similar suggestions to intensify depth of hypnosis and altered tone perception. When the auditory stimulus block ended, he asked the subject to close her eyes; and he administered a hypnotic reversal procedure, during which the subject opened her eyes.

**4) Post-hypnosis (PoH):** The experimenter instructed the subject to watch the video and delivered the last auditory stimulus block.

### 2.4 EEG recording

EEG was recorded using a 64-channel electrode cap and a BioSemi ActiveTwo Mk2^5^ with a 512 Hz sampling rate. The signal was 0–102.4 Hz band-pass filtered. Additional electrodes were attached to the tip of the nose and the left and right mastoids. Both vertical and horizontal eye movements were monitored, via electrodes below the left eye (VEOG) and at the right and left canthi (HEOG). Following BioSemi standard layout, the grounding electrode (CMS) was attached to the back of the head.

### 2.5 Methods of analysis

EEG data were preprocessed using BESA 7.0 software.^6^ First, signals were filtered: 0.53–45 Hz, 6 dB/octave, forward, for high pass; and 24 dB/octave, zero phase, for low pass. Ocular artefacts were corrected using the automatic PCA-artefact correction tool with default thresholds: 150 μV for HEOG amplitude and 250 μV for VEOG/blink. Automatic artefact correction did not work for one of the subjects under two of the conditions. Instead, a prominent eye blink was selected manually – from onset to offset visible at the frontal electrodes – to represent the artefact topography for the PCA process described above.

The data were re-referenced to the average of the mastoids. After visual inspection of the data, continuously noisy channels were interpolated for five subjects; for each subject, one out of the ten final electrodes used in the power analysis had to be interpolated from the original 64 electrodes. The last forty seconds of one subject's data in the post-hypnosis condition were lost due to emptying of the EEG amplifier battery.

The rest of the analysis was done in Matlab R2016a.^7^ First, data exported from BESA were epoched separately for each experimental condition. Power-spectral densities were calculated using the Spectopo function in EEGLAB toolbox^8^ (Delorme and Makeig, 2004), which uses Welch (1967) method for estimation and provides the power-spectral density in units of 10∗log10(μV^2^/Hz). The analysis window was four seconds, with a 50% overlap; with the sampling rate of 512 Hz, this resulted in a frequency resolution of 0.25 Hz.

Mean power-spectral density over each condition was calculated in nine frequency bands: delta (1–3.5 Hz), theta1 (3.5–6 Hz), theta2 (6–8 Hz), alpha1 (8–10 Hz), alpha2 (10–11.5 Hz), alpha3 (11.5–13 Hz), beta1 (13–19 Hz), beta2 (19–27 Hz), and gamma (27–45 Hz). Five electrodes from both hemispheres were included in analysis: Fp1, Fp2, F3, F4, C3, C4, P3, P4, O1, and O2. The similar frequency-band limits and electrode locations were used as in Kirenskaya et al. (2011). This was done to reduce variety in the analysis parameters – quite typical to EEG studies on hypnosis – which can complicate comparison of results between studies. This sample of 10 electrodes also represents well both the front-back and right-left scalp dimensions, commonly used in EEG studies. As the spatial resolution of the EEG is quite poor and the power differences between nearby electrodes are usually small, adding a larger number of electrodes in the analysis would mainly have increased the amount of redundant data (this was confirmed in a retrospective evaluation, where the mean powers over our 10 selected electrodes were found to be very close to those over all the 64 channels, indicating that our electrode selection represented well the general patterns in the data). Departing from Kirenskaya et al. (2011), only one gamma band was analyzed, with an upper limit of 45 Hz to avoid the 50-Hz noise from the local electrical network. The mean power-spectral density values were transferred to SPSS25^9^ for statistical analysis.

Nine 2 × 5×4 repeated-measure ANOVAs – one for each frequency band – were performed with *lateralization* (left- and right-side electrodes), *anteriority*/*posteriority* (frontopolar, frontal, central, parietal, and occipital electrode pairs) and *condition* (PrH, HY, SU, and PoH) as the within-subject factors. Measured power-spectral density at each electrode served as the dependent variable. Greenhouse-Geisser corrections for lack of sphericity were applied when appropriate; Bonferroni-corrected *post hoc* tests were conducted when necessary. Based on model diagnostics, the distributional assumptions of the ANOVA were met: although there were slight deviations from normality in the observed variables, the model residuals were normally distributed.

## 3 Results

**Figure 1:**
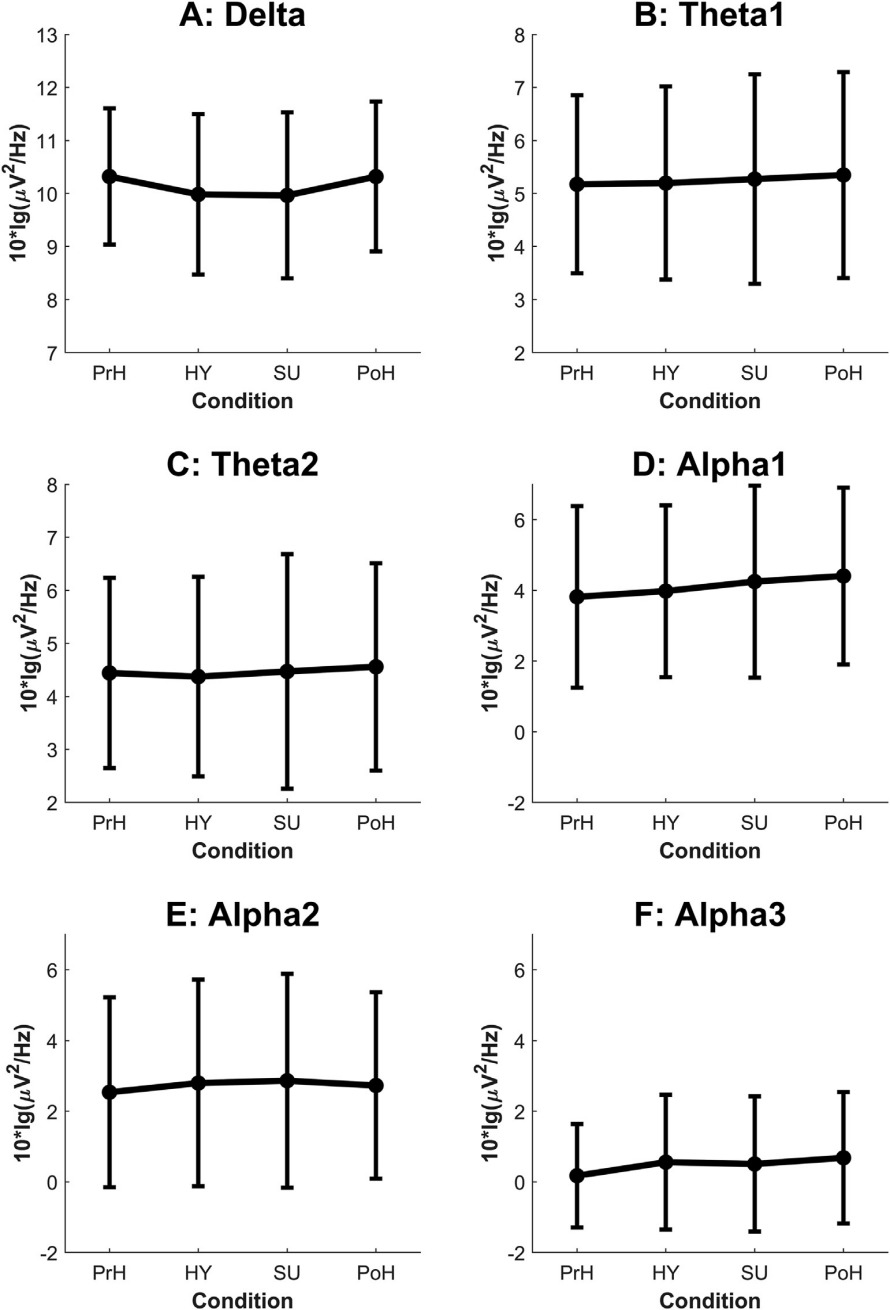
Mean oscillatory powers of the frequency ranges up to 14 Hz (panel A: delta, panel B: theta1, panel C: theta2, panel D: alpha1, panel E: alpha2, panel F: alpha3) in the four experimental conditions. PrH = pre-hypnosis, HY = neutral hypnosis, SU = hypnotic-suggestion, PoH = post-hypnosis. Error bars: 95% CI. *NB* the different scales in the panels.

**Figure 2:**
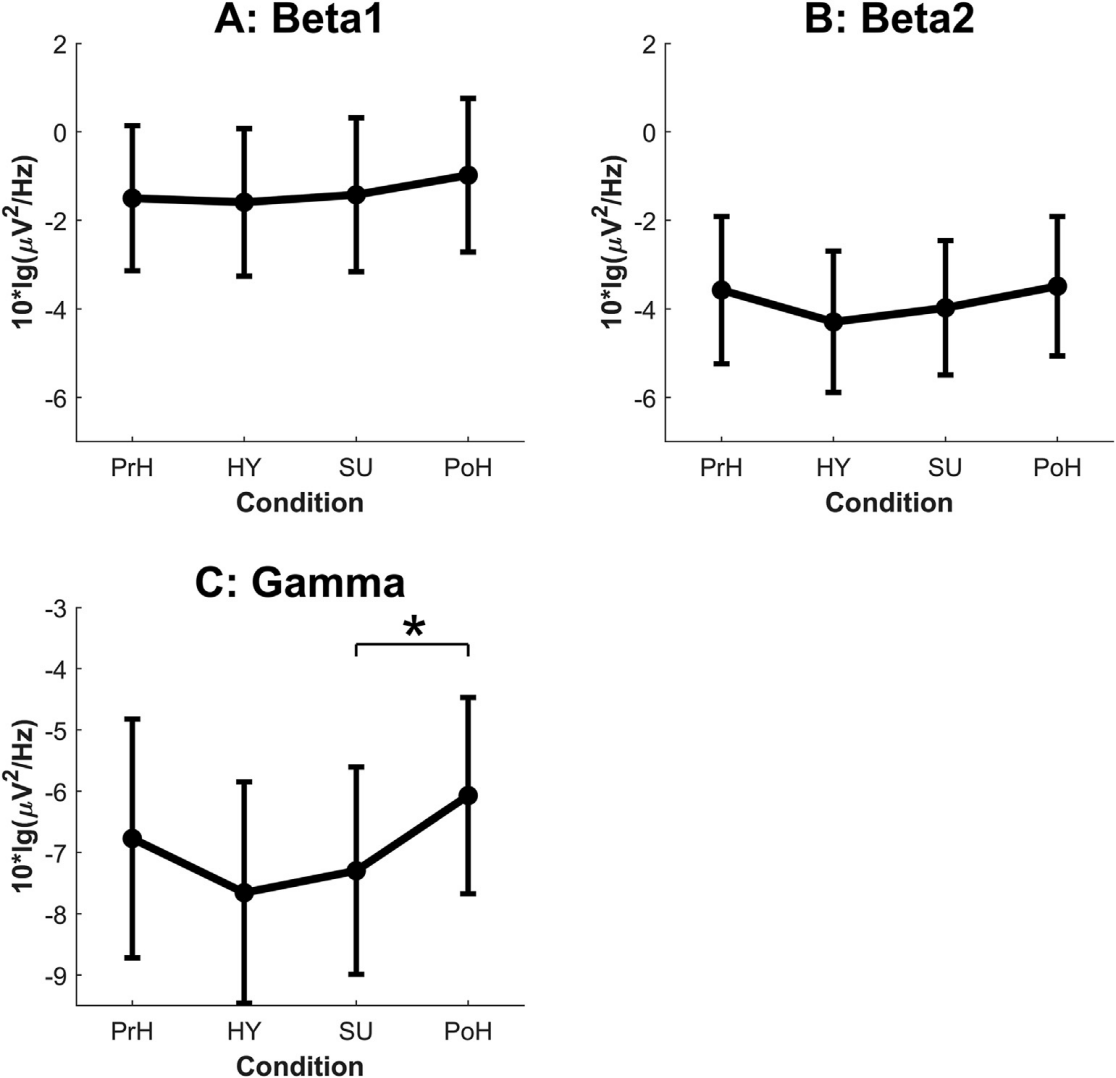
Mean oscillatory powers of the high-frequency ranges over 14 Hz (panel A: beta1, panel B: beta2, and panel C: gamma) in the four experimental conditions. PrH = pre-hypnosis, HY = neutral hypnosis, SU = hypnotic-suggestion, PoH = post-hypnosis. Error bars: 95% CI. ∗ = *p* < .05. *NB* the different scales in the panels.

The mean oscillatory powers in the lower (<14 Hz) frequency bands are presented in Figure 1 and those in the higher (>14 Hz) bands in Figure 2. No significant effects between conditions were found in the theta1 (F(3,24) = 0.20, *p* = .893, ηp^2^ = .03) or theta2 (F(3,24) = 0.16, *p* = .921, ηp^2^ = .02) ranges (Figure 1).

Condition was found to have a significant effect in the gamma band (F(3,24) = 3.63, *p* = .027, ηp^2^ = .31): *post hoc* tests with Bonferroni correction revealed a significant difference between SU and PoH (*p* = .029) and an almost significant difference between HY and PoH (*p* = .055). That means that the two hypnosis-related conditions exhibited less gamma power than the post-hypnosis condition (Figure 2). No significant effects of condition were found in the delta, alpha, or beta ranges (Figure 1). No significant difference in laterality was found in any frequency band.

**Figure 3:**
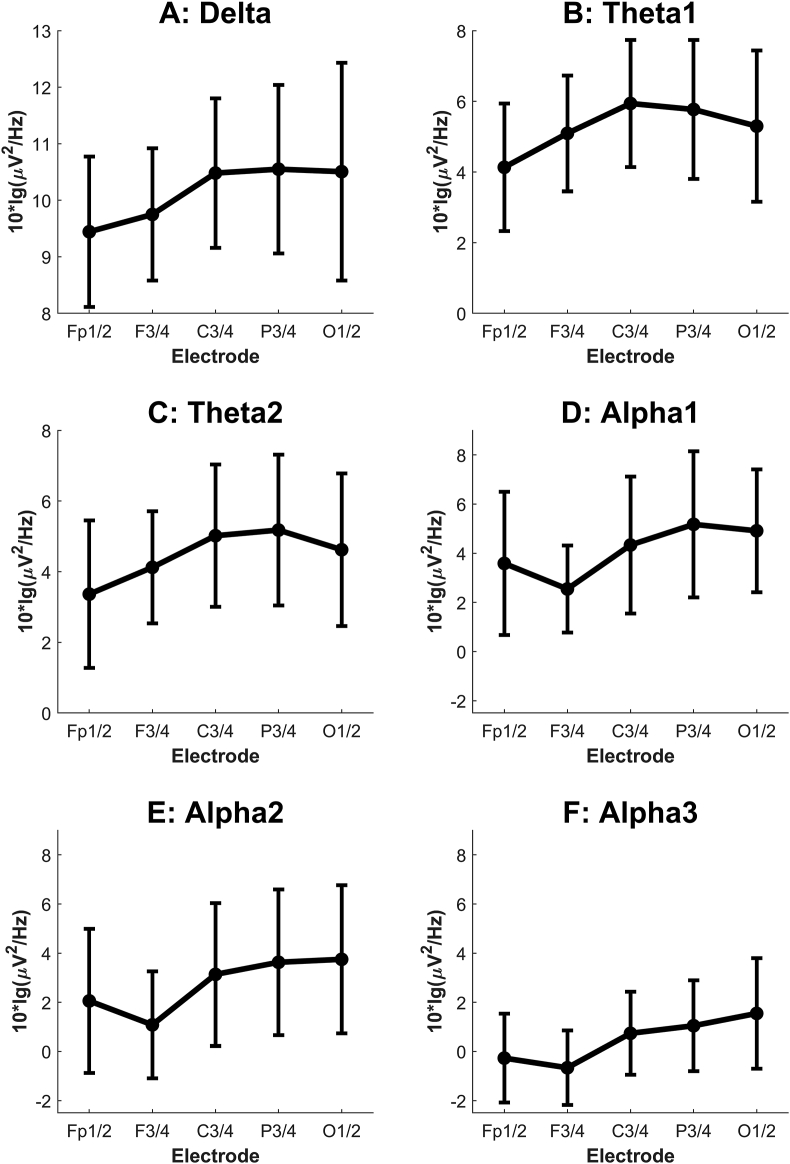
Mean oscillatory power in frequency ranges up to 14 Hz (panel A: delta, panel B: theta1, panel C: theta2, panel D: alpha1, panel E: alpha2, panel F: alpha3) in the electrodes on the anteroposterior dimension (Fp1/2, F3/4, C3/4, P3/4, O1/2) over the four conditions. Fp1/2 refers to the mean power calculated from electrodes Fp1 and Fp2, F3/4 refers to that of electrodes F3 and F4, etc. Error bars: 95% CI. *NB* the different scales in the panels.

Figure 3 shows the anteroposterior scalp distributions of the frequency bands. Mean power increased towards the central electrodes in the delta, theta1, and theta2 bands. In the alpha bands, power was lowest at the frontal electrodes and increased towards the posterior sites, reaching its highest values at the parietal (alpha1 and alpha2) or occipital (alpha2 and alpha3) electrodes. An effect in the anteroposterior scalp distribution (Figure 3) was found significant in the delta (F(4,32) = 4.71, *p* = .004, ηp^2^ = .37), theta1 (F(4,32) = 11.05, *p* < .001, ηp^2^ = .58), theta2 (F(4,32) = 7.55, *p* = .007, ηp^2^ = .49), alpha1 (F(4,32) = 8.78, *p* = .002, ηp^2^ = .52), alpha2 (F(4,32) = 13.99, *p* < .001, ηp^2^ = .64) and alpha3 (F(4,32) = 9.24, *p* = .004, ηp^2^ = .54) bands: i.e., systematically in oscillation bands lower than 14 Hz. No statistically significant interactions between the conditions and the anteroposterior dimension were found, implying that the anteroposterior power distribution remained stable over all conditions.

## 4 Discussion

The purpose of the study was to investigate whether oscillatory power in the various EEG frequency bands differs between pre-hypnosis (wake state), neutral hypnosis, hypnotic suggestion, and post hypnosis in highly hypnotizable subjects.

Working from a review on the relationship between brain oscillations and hypnosis (Jensen et al., 2015), we determined our three hypotheses. Hypothesis 1 held that theta power should increase under the neutral-hypnosis and hypnotic-suggestion conditions compared to the pre- and post-hypnosis conditions. No statistically significant difference in theta power between conditions was observed, however; visual inspection does not even suggest any trend (Figure 1).

Jensen et al. (2015) speculates that responding to hypnotic suggestions following hypnotic induction may be mediated by an increase in theta power, and theta oscillations may even be necessary for hypnotic response. However, the review's conclusions regarding theta power are mainly based on old EEG studies: namely, Sabourin et al. (1990) and Williams and Gruzelier (2001). The latter study actually found that highly hypnotizable subjects had the strongest theta activity in the post-hypnosis condition.

The present study differed from Sabourin et al. (1990) study with respect to the hypnotizability measure used: our standard twelve-item HGSHS:A vs. their ten-item modified HGSHS:A and individual Stanford Hypnotic Suggestibility Scale, Form C (SHSS:C; Weitzenhoffer and Hilgard, 1962). It differed as well with respect to sample size (nine vs. twelve) and subjects’ hypnotizability (highly hypnotizable vs. very highly hypnotizable).

Williams and Gruzelier (2001) study had one highly hypnotizable subject less than we had; and, during the EEG power measurements, subjects’ eyes were closed. The authors selected their eight subjects of high and eight of low hypnotizability from an initial 24 subjects, as assessed by the Barber Susceptibility Scale (BSS; Barber and Wilson, 1978). Due to the different hypnotizability assessment methods^10^ and criteria for inclusion, the samples of highly hypnotizable subjects probably differed between the studies; the heterogeneity of highly hypnotizable subjects will be discussed below.

Although the hypnotic induction used in all three studies included eye fixation, closing of the eyes, relaxation, and deepening of hypnosis, slight procedural differences could be noted. Williams and Gruzelier (2001) used a longer counting process to deepen hypnosis then proceeded to give further deepening suggestions via guided imagery. Sabourin et al. (1990) used tape-recorded induction followed by deepening suggestions that are, unfortunately, not described in detail.

Our theta results are in line with more recent studies using multichannel EEG that have found no spectral-power changes in any frequency band between wake state and hypnosis (Jamieson and Burgess, 2014; White et al., 2008). One could argue that our sample size was too small for detecting such changes; however, as mentioned, there was not even a trend in the theta bands in the predicted direction (Figure 1). Moreover, the effect sizes in the theta bands were small (theta1: ηp^2^ = .03, theta2: ηp^2^ = .02). It strikes us as unlikely that adding even a substantial number of subjects would have changed the results.

One could argue that our subjects might not have been in deep enough hypnosis to reveal theta-power effects. Under the neutral-hypnosis condition (HY), subjects' subjective mean hypnotic-depth evaluations were 5.8 (sd = 1.7) on a scale of 0–10; under the hypnotic-suggestion condition (SU), they were 5.7 (sd = 2.7); see Hiltunen et al. (2019). We chose the characteristics of our auditory and visual stimuli (tone intensity, video contents) in a way that would cause as little disturbance as possible to the subject's hypnotic state. Keeping the limitations of subjective hypnotic-depth evaluations in mind (Radtke & Spanos, 1981, 1982), four out of nine subjects reported that opening their eyes influenced their hypnotic state, and about half felt that their hypnosis had been deeper in the hypnotizability group assessment than the actual experiment, as discussed in Hiltunen et al. (2019). This suggests that, at least in some subjects, experienced deeper hypnosis might have been possible with extra hypnosis-deepening suggestions or by having subjects keep the eyes closed.

That said, alertness and attentiveness during hypnosis – which having the eyes open may provoke – have not been found to influence hypnotic responsiveness (Banyai and Hilgard, 1976). Comparing our subjects’ alertness or depth of hypnosis with those of Sabourin et al. (1990) or Williams and Gruzelier (2001) cannot be done reliably. They used different methods for evaluating hypnotic state: namely, testing based on motor, hallucinatory, and hypnotic dreaming suggestions, versus objective and subjective BSS scores. In any case, our results suggest that theta power cannot be used as a neural marker of hypnotic state for highly hypnotizable subjects, despite our initial expectations.

It is important to report and discuss results where the null hypothesis could not be rejected (Fanelli, 2010; Ferguson and Heene, 2012) – as evidenced by the replication crisis in psychology (Maxwell et al., 2015; Shrout and Rodgers, 2018) and cognitive neuroscience (see e.g. Huber et al., 2019). This is especially important where current theories may be based on the results of too few or conflicting studies.

We hypothesized (Hypothesis 2) a change in gamma power between the non-hypnosis (PrH, PoH) and hypnosis (HY, SU) conditions. The hypothesis was partially confirmed. Interestingly, the statistically significant change was not between the pre-hypnosis (PrH) and hypnosis (HY, SU) conditions but between the hypnotic-suggestion (SU) and post-hypnosis (PoH) conditions: namely, gamma power decreased in the SU condition compared to PoH. The mean gamma powers under both hypnotic conditions were highly similar (Figure 2), while the decrease of gamma power in HY compared to PoH almost reached statistical significance. Visual inspection revealed a trend such that gamma power decreased from PrH to the HY and SU conditions then increased to the PoH condition.

According to the most recent American Psychological Association definition (Elkins et al., 2015), hypnosis is characterized by reduced peripheral awareness.^11^ The lower gamma power in our hypnotic conditions may be related to hypnosis’ ability to decrease awareness of task-irrelevant sensory stimuli such as our short tone beeps played in the background. The observed trend of gamma reduction under hypnosis is consistent with the results of a case study with a single highly hypnotizable subject (Hinterberger et al., 2011). That said, highly hypnotizable subjects in another study revealed moderately strong *positive* correlations between hypnotic depth and power in the gamma as well as beta2 and beta3 bands (Cardeña et al., 2013) – contrary to the reduced perceptual awareness hypothesis. Given the contradictory results and keeping in mind the maxim that correlation does not equal causation, we doubt whether a deeper level of hypnosis would have increased gamma power in the hypnosis conditions and so eliminated the power difference between them and the PoH condition.

Interestingly in the present study, the gamma power in PoH did not just return to the wake-state baseline (PrH) but was enhanced in comparison. So far as we know, no such increase in gamma power in a post-hypnosis condition has ever been reported previously. In the aforementioned study of a hypnotic virtuoso (Fingelkurts et al., 2007), the EEG spectral pattern did not return to pre-hypnosis levels directly after hypnosis. On that basis, one would have expected larger differences to be observed between PrH and HY/SU conditions. However, since only a few earlier spectral-power studies have included post-hypnosis conditions, it is difficult to speculate on the reasons for the observed gamma enhancement.

Our third hypothesis was not confirmed at all: i.e., we found no statistically significant gamma-power differences between the HY and SU conditions. Jensen et al. (2015) speculates that the way hypnotic suggestions are implemented might result in gamma-power changes. Since auditory suggestions similar to those used in the present study have not been employed previously, direct comparison to earlier studies is impossible. Our hypnotic suggestion effects under SU might have been too weak to be manifested in power differences between HY and SU. That said, the difference between HY and PoH did not reach statistical significance whereas that between SU and PoH did, so the gamma power may have been influenced by our hypnotic suggestion for altering tone perception (Hiltunen et al., 2019, p. 199):

They are just in the background as if they are meaningless and muffled… All the beeping sounds sound exactly the same in pitch… without meaning, soft in the background, with a similar pitch….

The suggestion may have decreased subjects’ awareness of task-irrelevant auditory sensory stimuli in the SU condition compared to HY. Although the absolute level of mean gamma power was lowest in HY (Figure 2), it was more consistently reduced (i.e., with a smaller standard deviation) in SU.

One could question whether our neutral-hypnosis condition (HY) was truly neutral. Even there, participants were asked to perform certain tasks such as to keep their eyes open, watch the video, and try to avoid excessive blinking. Such “suggestions” are difficult to avoid in this kind of experiment.

Hypnosis-related scalp-distribution changes have been reported in various power bands in earlier studies with highly hypnotizable subjects: e.g., De Pascalis (1993) reports a decrease in alpha1 and alpha2 amplitudes in posterior areas during hypnotic dream and age regression, compared to neutral hypnosis; Fingelkurts et al. (2007) report decreased delta and increased beta power in frontal areas during hypnosis, compared to baseline, in a study with a hypnotic virtuoso; Graffin et al. (1995) report increased theta power in posterior scalp areas and increased alpha activity across all areas during hypnotic induction. The present study found significant effects in the anteroposterior scalp distribution in all oscillation bands under 14 Hz: i.e., the delta, theta, and alpha ranges. However, no statistically significant interactions were observed between the anteroposterior electrode dimension and the conditions, indicating that the anteroposterior power distribution remained stable across conditions. Still, comparing our results to the earlier literature is challenging, since the experimental conditions differed so much between studies.

We found no lateralization differences in the oscillatory power bands. A study by Crawford et al. (1996) did find a lateralization effect, albeit with suggestions very different from ours: during hypnosis, self-generated sad emotions from re-experienced past events produced less low alpha activity in the right parietal area compared to happy emotions. Some older studies (e.g., De Pascalis, 1993; Sabourin et al., 1990) report small lateralized power changes, but they have not been confirmed by later studies (Jamieson and Burgess, 2014; White et al., 2008). The type of suggestion can influence lateralization: e.g., left-arm levitation suggestions have been shown to produce effects in the opposite hemisphere (Hinterberger et al., 2011). We deliberately chose to employ hemisphere-neutral suggestions for hypnotic induction (HY) and the perception-altering suggestion (SU); visual stimuli were presented equally to both visual fields and auditory stimuli to both ears.

It is worth speculating on possible further reasons for the apparent discrepancies between the results of the present study and previous ones. These include limitations inherent in EEG (see e.g. Jensen et al., 2015), which must be considered when interpreting gamma readings. Higher-frequency oscillations have amplitudes orders of magnitude smaller than the slower theta or alpha activities. Higher-frequency oscillations typically reflect more regional brain activity and summate less well on the scalp. Previous studies have shown that distinct spectral changes appear immediately after suggestions (Halsband and Wolf, 2019). The brain activity observed in the fast EEG oscillations is probably condition and suggestion specific. Testing hypotheses related to high frequencies is generally more challenging than testing those related to lower frequencies (Jensen et al., 2015).

Theta power has fairly consistently been shown to reflect hypnotizability when highly hypnotizable subjects are compared to low hypnotizable ones in wake state (Graffin et al., 1995; Kirenskaya et al., 2011; Vanhaudenhuyse et al., 2014). The results of the present study suggest that the hypnotic state may not produce any further increase in theta power among highly hypnotizable subjects. If such an effect exists, it is likely to be weak and easily confounded with a wide range of experimental (choice of induction, suggestions, tasks, etc.) and individual-specific sources of variation.

Highly hypnotizable subjects are not a homogenous group (Terhune, 2015). One subtype has been found to be more responsive to positive or negative suggestions for hallucination and experience greater involuntariness. Another has displayed superior visual object imagery (Terhune et al., 2011). Under hypnosis, highly hypnotizable subjects’ imagery has been found to correlate positively with gamma-power heterogeneity and negatively with alpha1-power heterogeneity (Cardeña et al., 2013). Subjects may use different strategies to implement suggestions (Oakley and Halligan, 2013).

Difficulties controlling cognitive processes between conditions may be a further source of variability in EEG-based studies (White et al., 2008). The task we asked our subjects to perform was quite stationary: watching a peaceful video while gentle beeps sounded in the background. Given such a task, we could not do much to guide subjects' cognitive processes while the task was under way: e.g., whether subjects concentrated continuously on the video watching or sometimes allowed their minds to wander. Subjects’ spontaneous comments after the experiment indicated variable experiences: for two subjects, video watching was easier in hypnosis; two had the opposite experience; one reported no difference.

Our subjects had their eyes open under all conditions. In our original ERP study (Hiltunen et al., 2019), where the EEG data for the present study were recorded, we were forced to have our subjects' eyes open to prevent excessive alpha activity contaminating the ERPs. It is well known that closing the eyes typically enhances alpha power (Graffin et al., 1995; Lubar et al. 1991). If our subjects’ eyes had been closed, we would have expected higher alpha power under all conditions. Two previous hypnosis studies conducted parts of their experiments both eyes open and eyes closed. In wake state with eyes closed, highly hypnotizable subjects have exhibited higher alpha2 (De Pascalis, 1993) and theta2 and alpha1 amplitudes (De Pascalis et al., 1998) compared to when they had their eyes open. Sabourin et al. (1990) found that highly hypnotizable subjects in wake state with eyes open showed significantly more overall beta power in the left than the right hemisphere, but not in conditions where they had their eyes closed. Geller et al. (2014) observed in an electrocorticogram recording that eye closure caused widespread low-frequency (i.e., delta, theta, alpha, and beta) power increase and focal gamma attenuation.

Other methodological differences between the various brain-oscillation studies exist. We used a 4-s time window to achieve a “good enough” frequency resolution of 0.25 Hz. Although previous studies (e.g., De Pascalis, 1993) have typically used similar resolution, some studies (e.g., Cardeña et al., 2013) have used 0.5 Hz; while in other cases (e.g., Kirenskaya et al., 2011), the resolution has not been reported. We used a sliding-window technique in our analysis, windows overlapping 50% over the course of each condition (about six minutes) with the power values averaged for each band. The time period on which earlier studies made their power estimates varied from twenty seconds to five minutes (Cardeña et al., 2013; De Pascalis, 1993; Graffin et al. 1995; Kirenskaya et al., 2011; Sabourin et al., 1990; White et al., 2008; Williams and Gruzelier, 2001). By using the sliding-window technique to determine spectral power over the entire duration of the conditions, we acquired a reasonably stable, mean representation of the oscillatory power for each condition such that short lapses in subjects’ attention would not have affected results.

EEG methodology has been developing rapidly over the last decades. Modern multichannel EEG systems with active electrodes are typically less sensitive to artefacts (Mathewson et al., 2017) at the same time as enabling better artefact correction: e.g., component analysis for removing eye blinks.

The precise upper and lower limits of the EEG frequency bands studied have varied widely between studies: it is hard to find two studies where all the thresholds have been the same. This makes it far from clear the extent to which frequency bands from different studies include analogous neural phenomena. To make our results more comparable at least to one recent multichannel study (Kirenskaya et al., 2011), we used the same frequency bands and electrode montage.

The results from studies exploring the effects of hypnosis and hypnotic suggestion on EEG spectral power have been, as noted in the beginning of this paper, heterogenous. This may be caused by the great variability between studies regarding intracerebral source locations, EEG dimensionality, measurement techniques, and methods of analysis (Halsband and Wolf, 2019); as well as by differences in hypnotizability estimates, experimental stimuli, tasks, inductions, and suggestions. In their study (which found no hypnosis-related spectral-power differences), Jamieson and Burgess (2014) conclude that, if hypnosis-specific patterns of EEG band power existed, they would have been found long ago, given just how many studies have measured EEG during hypnosis. Changes in EEG spectral power during hypnosis in highly hypnotizable subjects may be well in the range seen in normal, non-hypnosis conditions.

What are the strengths of our study? We included pre-hypnosis, neutral-hypnosis, hypnotic-suggestion and post-hypnosis conditions in the same study, allowing better comparability between conditions. To get information on possible carry-over effects, inclusion of a post-hypnosis condition should be considered a golden standard for future hypnosis studies. Subjects had their eyes open under all conditions, so that excessive alpha activity and possible effects on the other EEG frequency bands (Geller et al., 2014) were avoided.

Naturally, our study has limitations, which should be considered when interpreting its results. Since we reused the EEG data from our earlier ERP study, we were not able to optimize the experiment for purposes of the present study. It would have been interesting to include measurements without auditory or visual stimuli and different types of hypnotic suggestions that might have functioned better than the rather demanding auditory one we used.

Our experiment included only highly hypnotizable subjects. Future studies should include subjects of medium and low hypnotizability, given that different spectral-power patterns have been observed between subjects of high and low hypnotizability (Cardeña et al., 2013; Williams and Gruzelier 2001). Our study measured subjects’ hypnotizability just once, using HGSHS:A. An individual assessment (e.g., SHSS:C) in addition to a group variant would give a more precise hypnotizability estimate.^12^

In conclusion, we did not find evidence for the proposal of an increase in theta power as a marker of hypnotic state in highly hypnotizable subjects. However, we did find suggestive changes in the gamma bands between hypnosis (HY, SU) and post-hypnosis conditions. Further studies are needed to confirm the findings and reach stronger conclusions about the theoretical significance of these effects.

## Declarations

### Author contribution statement

Seppo Hiltunen: Conceived and designed the experiments; Performed the experiments; Contributed reagents, materials, analysis tools or data; Wrote the paper.

Maria Karevaara: Analyzed and interpreted the data; Contributed reagents, materials, analysis tools or data; Wrote the paper.

Maarit Virta: Conceived and designed the experiments; Performed the experiments; Wrote the paper.

Tommi Makkonen: Conceived and designed the experiments; Analyzed and interpreted the data; Contributed reagents, materials, analysis tools or data; Wrote the paper.

Sakari Kallio, Petri Paavilainen: Conceived and designed the experiments; Wrote the paper.

### Funding statement

This research did not receive any specific grant from funding agencies in the public, commercial, or not-for-profit sectors.

### Data availability statement

The authors do not have permission to share data.

### Declaration of interests statement

The authors declare no conflict of interest.

### Additional information

No additional information is available for this paper.

## Footnotes

1 For further details of the recruitment process and subjects' background information, see Hiltunen et al. (2019).

2 OutpostFX AB, http://www.outpostfx.com.

3 For further details, see Hiltunen et al. (2019).

4 For further details on the procedure and the contents of the hypnotic suggestions, see Hiltunen et al. (2019).

5 BioSemi B.V., The Netherlands, http://www.biosemi.com.

6 BESA GmbH, Germany.

7 The MathWorks, USA.

8 Version 14.1.2: http://www.sccn.ucsd.edu/eeglab.

9 IBM, USA.

10 For a study comparing HGSHS:A and BSS; Polczyk (2016).

11 See also the conclusions of the review conducted by Vanhaudenhuyse et al. (2014).

12 For further limitations, see Hiltunen et al. (2019).
